# Supplementary material for: Deciphering the Regulatory Potential of Antioxidant and Electron-Shuttling Bioactive Compounds in Oolong Tea
Source: Biology (Basel). 2025 Apr 28;14(5):487. doi: 10.3390/biology14050487 (PMC12109060; doi:10.3390/biology14050487)
Supplement: Supplementary file 1 [file biology-14-00487-s001.zip › biology-3526008-supplementary.pdf]

Deciphering the regulatory potential of antioxidant and electron-shuttling bioactive compounds in Oolong tea

Regineil A. Ferrer [1,2,†], Bor-Yann Chen [3,†], Jon Patrick T. Garcia [1,2], Christine Joyce F. Rejano [1,2], Po-Wei Tsai [4], Chung-Chuan Hsueh [3], and Lemmuel L. Tayo [5,*]

[1] School of Chemical, Biological, and Materials Engineering and Sciences, Mapúa University, Manila 1002, Philippines
[2] School of Graduate Studies, Mapúa University, Manila 1002, Philippines
[3] Department of Chemical and Materials Engineering, National I-lan University, I-lan 260, Taiwan
[4] Department of Food Science, National Taiwan Ocean University, Keelung 202, Taiwan
[5] Department of Biology, School of Health Sciences, Mapúa University, Makati 1200, Philippines
[†] These authors contributed equally to this work
[*] Correspondence: lltayo@mapua.edu.ph

Table S1. Electrochemical profiles of OT extracts using CV over 50 cycles at different pH conditions.

Cycle	OTL-W			OTL-E			OTS-W			OTS-E		
	pH 4.53	pH 8.24	pH 9.50	pH 4.20	pH 8.30	pH 9.50	pH 4.10	pH 8.18	pH 9.50	pH 4.31	pH 8.40	pH 9.50
5	136.159	152.510	143.233	51.527	112.267	31.778	121.868	109.007	63.292	41.761	108.700	87.466
10	141.706	139.554	95.640	52.709	63.686	12.810	128.856	81.306	52.410	47.435	30.814	66.221
15	142.710	118.887	74.355	54.850	24.556	12.606	124.841	63.820	46.787	50.7161	21.087	54.313
20	146.690	108.623	63.003	55.879	13.048	13.632	113.376	51.416	44.616	54.757	14.775	46.096
25	146.905	99.909	52.707	54.288	13.387	12.819	90.087	36.729	37.969	52.297	15.299	35.453
30	138.598	92.729	31.904	52.448	13.618	13.632	67.156	20.351	37.671	52.537	15.459	27.203
35	130.350	85.208	32.128	50.850	14.006	13.416	48.955	20.108	31.956	51.147	15.552	23.214
40	121.487	80.611	28.553	47.380	14.181	13.126	30.183	19.992	29.754	50.878	16.244	20.611
45	110.549	74.823	28.301	41.954	14.178	14.028	16.422	19.973	26.877	48.362	16.014	20.488
50	99.986	73.296	27.747	37.156	14.277	14.223	14.359	20.870	23.137	45.215	16.617	18.626

Table S2. Screening of the bioactive compounds in OT with electron-shuttling properties using web servers SwissADME and ADMETLAB 3.0.

Compounds	PubChem ID	SwissADME										ADMETLAB 3.0									Electron Shuttle	
		Physicochemical Properties						Druglikeness			Pharmaco-kinetics	Physicochemical properties						Druglikeness		Pharmaco-kinetics	Ortho	Para
		MW	nRot	nHA	nHD	iLOGP	TPSA	Lipinski	Veber	BA	GI abs	MW	nRot	nHA	nHD	logP	TPSA	Lipinski	Veber	hia		
(-)-Catechin	73160	290.27	1	6	5	1.36	110.38	Passed	Passed	0.55	High	290.08	1	6	5	0.938	110.38	Passed	Passed	0.002642	1	0
(-)-Epicatechin	72276	290.27	1	6	5	1.47	110.38	Passed	Passed	0.55	High	290.08	1	6	5	1.094	110.38	Passed	Passed	0.016011	1	0
(-)-Epigallocatechin	72277	306.27	1	7	6	0.98	130.61	Passed	Passed	0.55	High	306.07	1	7	6	0.794	130.61	Passed	Passed	0.025356	2	0
(-)-Gallocatechin	9882981	306.27	1	7	6	1.24	130.61	Passed	Passed	0.55	High	306.07	1	7	6	0.500	130.61	Passed	Passed	0.005632	2	0
(-)-Icariside B5	14135399	388.45	6	8	5	1.85	136.68	Passed	Passed	0.55	High	388.21	6	8	5	0.163	136.68	Passed	Passed	0.005188	2	0
(+)-Catechin	9064	290.27	1	6	5	1.33	110.38	Passed	Passed	0.55	High	290.08	1	6	5	1.173	110.38	Passed	Passed	0.016103	1	0
(+)-Epicatechin	182232	290.27	1	6	5	1.37	110.38	Passed	Passed	0.55	High	290.08	1	6	5	0.842	110.38	Passed	Passed	0.001434	1	0
(+)-Gallocatechin	65084	306.27	1	7	6	1.47	130.61	Passed	Passed	0.55	High	306.07	1	7	6	0.877	130.61	Passed	Passed	0.086205	2	0
1-(3,4,5-trihydroxyphenyl) ethanone	36586	168.15	1	4	3	0.61	77.76	Passed	Passed	0.55	High	168.04	1	4	3	0.928	77.76	Passed	Passed	0.011835	2	0
Ascorbic acid	54670067	176.12	2	6	4	-0.31	107.22	Passed	Passed	0.56	High	176.03	2	6	4	-1.707	107.22	Passed	Passed	0.219913	1	0
Caffeic acid	689043	180.16	2	4	3	0.97	77.76	Passed	Passed	0.56	High	180.04	2	4	3	1.190	77.76	Passed	Passed	0.301189	1	0
Catechol	289	110.11	0	2	2	1.13	40.46	Passed	Passed	0.55	High	110.04	0	2	2	1.025	40.46	Passed	Passed	0.139801	1	0
Chakanoside I	91412699	298.29	5	7	4	1.1	116.45	Passed	Passed	0.55	High	298.11	5	7	4	-0.299	116.45	Passed	Passed	0.267714	2	0
Cyanidin	128861	287.24	1	6	5	-2.62	114.29	Passed	Passed	0.55	High	287.06	1	6	5	0.930	112.45	Passed	Passed	0.029234	1	0
Delphinidin	68245	338.7	1	7	6	-5.53	134.52	Passed	Passed	0.55	High	303.05	1	7	6	0.423	132.68	Passed	Passed	0.068387	2	0
Dihydroquercetin	439533	304.25	1	7	5	0.71	127.45	Passed	Passed	0.55	High	304.06	1	7	5	0.932	127.45	Passed	Passed	0.065961	1	0
Fisetin	5281614	286.24	1	6	4	1.5	111.13	Passed	Passed	0.55	High	286.05	1	6	4	1.621	111.13	Passed	Passed	0.36284	1	0
Gallic acid	370	170.12	1	5	4	0.21	97.99	Passed	Passed	0.56	High	170.02	1	5	4	0.692	97.99	Passed	Passed	0.017095	2	0
Leucocyanidin	71629	306.27	1	7	6	1.19	130.61	Passed	Passed	0.55	High	306.07	1	7	6	0.353	130.61	Passed	Passed	0.033822	1	0
Luteolin	5280445	286.24	1	6	4	1.86	111.13	Passed	Passed	0.55	High	286.05	1	6	4	2.247	111.13	Passed	Passed	0.014605	1	0
Quercetin	5280343	302.24	1	7	5	1.63	131.36	Passed	Passed	0.55	High	302.04	1	7	5	1.448	131.36	Passed	Passed	0.133589	1	0
Tricetinidin	11199650	287.24	1	6	5	-2.42	114.29	Passed	Passed	0.55	High	287.06	1	6	5	0.369	112.45	Passed	Passed	0.016088	2	0

Table S3. Top 5 GO terms for BP, CC, and MF across BC subtypes in response to OT bioactive compounds.

BC subtype	Category	Term	Description	Fold Enrichment	FDR
OTxLaBC_Down	BP	GO:0010469	regulation of signaling receptor activity	96.735	0.0219
		GO:1904385	cellular response to angiotensin	72.551	0.0344
		GO:1904645	response to amyloid-beta	72.551	0.0344
		GO:0060749	mammary gland alveolus development	64.490	0.0419
		GO:2000811	negative regulation of anoikis	61.096	0.0419
	CC	GO:0043197	dendritic spine	12.640	0.0115
		GO:0031012	extracellular matrix	8.532	0.0358
		GO:0043025	neuronal cell body	7.584	0.00986
		GO:0043005	neuron projection	7.492	0.0180
		GO:0030424	axon	6.467	0.0315
	MF	GO:0043560	insulin receptor substrate binding	87.348	0.0105
		GO:0004175	endopeptidase activity	25.234	0.00230
		GO:0004713	protein tyrosine kinase activity	18.198	0.00457
		GO:0004222	metalloendopeptidase activity	16.457	0.00551
		GO:0016301	kinase activity	11.025	0.00514
OTxLaBC_Up	BP	GO:0071395	cellular response to jasmonic acid stimulus	435.309	0.00118
		GO:0044597	daunorubicin metabolic process	193.471	0.00633
		GO:0044598	doxorubicin metabolic process	174.124	0.00659
		GO:0042448	progesterone metabolic process	102.426	0.0148
		GO:0006693	prostaglandin metabolic process	79.147	0.0223
	CC	GO:0043235	receptor complex	30.034	1.36E-10
		GO:0009986	cell surface	6.545	0.0267
		GO:0005829	cytosol	2.066	0.0267
	MF	GO:0018636	phenanthrene 9,10-monooxygenase activity	567.765	4.64E-04
		GO:0097621	monoamine oxidase activity	378.510	0.0446
		GO:0047115	trans-1,2-dihydrobenzene-1,2-diol dehydrogenase activity	378.510	0.0446
		GO:0047718	indanol dehydrogenase activity	378.510	0.0446
		GO:0047086	ketosteroid monooxygenase activity	283.882	0.00139
OTxLbBC_Down	BP	GO:0071492	cellular response to UV-A	97.855	0.0161
		GO:0160049	negative regulation of cGAS/STING signaling pathway	89.700	0.0168
		GO:0010469	regulation of signaling receptor activity	89.700	0.0168
		GO:1904355	positive regulation of telomere capping	79.733	0.00148
		GO:1904645	response to amyloid-beta	67.275	0.0264
	CC	GO:0031012	extracellular matrix	9.494	0.00911
		GO:0032991	protein-containing complex	6.107	4.78E-04
		GO:0005813	centrosome	5.094	0.00849
		GO:0005654	nucleoplasm	2.356	8.97E-04
		GO:0005829	cytosol	2.151	4.15E-04
	MF	GO:0019899	enzyme binding	8.491	2.89E-04
		GO:0019901	protein kinase binding	6.992	2.89E-04
		GO:0005524	ATP binding	5.239	3.17E-09
		GO:0008270	zinc ion binding	5.092	2.13E-04
		GO:0003682	chromatin binding	4.698	0.0471

OTxLbBC_Up	BP	GO:0071395	cellular response to jasmonic acid stimulus	314.904	0.00257
		GO:0044597	daunorubicin metabolic process	139.957	0.0108
		GO:0044598	doxorubicin metabolic process	125.962	0.0127
		GO:0006693	prostaglandin metabolic process	76.340	0.00190
		GO:0042448	progesterone metabolic process	74.095	0.0298
	CC	GO:0043235	receptor complex	25.677	7.58E-12
		GO:0045121	membrane raft	12.637	0.00363
		GO:0030424	Axon	9.356	0.00134
		GO:0009986	cell surface	6.088	0.00363
		GO:0005829	cytosol	1.967	0.00764
	MF	GO:0018636	phenanthrene 9,10-monooxygenase activity	410.723	9.61E-04
		GO:0047086	ketosteroid monooxygenase activity	205.361	0.00383
		GO:0047023	androsterone dehydrogenase activity	154.021	0.00508
		GO:0047044	androstan-3-alpha,17-beta-diol dehydrogenase activity	136.908	0.00508
		GO:0016655	oxidoreductase activity, acting on NADPH, quinone or similar compound as acceptor	136.908	0.00508
OTxHer2BC_Down	BP	GO:0071492	cellular response to UV-A	80.328	0.0218
		GO:1904645	response to amyloid-beta	73.634	0.00181
		GO:0160049	negative regulation of cGAS/STING signaling pathway	73.634	0.0225
		GO:0010469	regulation of signaling receptor activity	73.634	0.0225
		GO:0033628	regulation of cell adhesion mediated by integrin	67.970	0.0248
	CC	GO:0000922	spindle pole	10.676	0.0268
		GO:0031012	extracellular matrix	9.093	0.00301
		GO:0030424	axon	5.743	0.0268
		GO:0005813	centrosome	5.575	4.75E-04
		GO:0005925	focal adhesion	5.0166	0.0462
	MF	GO:0004712	protein serine/threonine/tyrosine kinase activity	36.015	0.00410
		GO:0004175	endopeptidase activity	26.891	1.28E-05
		GO:0048156	tau protein binding	26.802	0.00922
		GO:0004222	metalloendopeptidase activity	17.538	1.00E-04
		GO:0016301	kinase activity	13.986	2.49E-06
OTxHer2BC_Up	BP	GO:0071395	cellular response to jasmonic acid stimulus	302.051	0.00264
		GO:0044597	daunorubicin metabolic process	134.245	0.0118
		GO:0044598	doxorubicin metabolic process	120.820	0.0139
		GO:0097242	amyloid-beta clearance	92.939	0.0214
		GO:0042448	progesterone metabolic process	71.071	0.0335
	CC	GO:0043235	receptor complex	22.734	3.76E-10
		GO:0045121	membrane raft	10.101	0.0308
		GO:0030424	axon	8.974	0.00161
		GO:0009897	external side of plasma membrane	6.955	0.0190
		GO:0043025	neuronal cell body	6.766	0.0325
	MF	GO:0016301	kinase activity	9.562	0.0208
		GO:0008270	zinc ion binding	4.397	0.00454
		GO:0005524	ATP binding	3.835	5.55E-04
		GO:0042802	identical protein binding	2.722	0.0321

		GO:0005515	protein binding	1.297	0.0264
OTxTNBC_Down	BP	GO:0007077	mitotic nuclear membrane disassembly	108.827	0.0123
		GO:0071492	cellular response to UV-A	79.147	0.0203
		GO:0010469	regulation of signaling receptor activity	72.551	0.0214
		GO:1904355	positive regulation of telomere capping	64.490	0.00213
		GO:2000811	negative regulation of anoikis	61.096	0.00231
	CC	GO:0005876	spindle microtubule	28.573	0.00913
		GO:0000307	cyclin-dependent protein kinase holoenzyme complex	27.924	0.00913
		GO:0000922	spindle pole	10.519	0.0240
		GO:0031012	extracellular matrix	7.679	0.0222
		GO:0005813	centrosome	6.409	6.82E-06
	MF	GO:0004693	cyclin-dependent protein serine/threonine kinase activity	39.156	0.00381
		GO:0004715	non-membrane spanning protein tyrosine kinase activity	24.685	0.0108
		GO:0004175	endopeptidase activity	22.711	2.15E-04
		GO:0106310	protein serine kinase activity	15.513	3.34E-15
		GO:0004674	protein serine/threonine kinase activity	14.863	3.69E-15
OTxTNBC_Up	BP	GO:0071395	cellular response to jasmonic acid stimulus	296.010	0.00324
		GO:0044597	daunorubicin metabolic process	131.560	0.0148
		GO:0044598	doxorubicin metabolic process	118.404	0.0172
		GO:0030518	nuclear receptor-mediated steroid hormone signaling pathway	98.670	0.0220
		GO:0097242	amyloid-beta clearance	91.080	0.0244
	CC	GO:0043235	receptor complex	14.853	1.22E-04
		GO:0030424	axon	7.695	0.0171
		GO:0009897	external side of plasma membrane	6.816	0.0218
	MF	GO:0018636	phenanthrene 9,10-monooxygenase activity	386.080	0.00115
		GO:0043559	insulin binding	231.648	0.00283
		GO:0047086	ketosteroid monooxygenase activity	193.040	0.00328
		GO:0047023	androsterone dehydrogenase activity	144.780	0.00457
		GO:0016655	oxidoreductase activity, acting on NAD(P)H, quinone or similar compound as acceptor	128.693	0.00457

Table S4. Binding affinities of selected inhibitors and pre-bound ligands to breast cancer-associated hub proteins.

Gene name<br>PDB ID	ESR1<br>4MG6	HSP90AB1<br>7ULJ	IGF1R<br>3LW0	MAPK1<br>8U8J	PIK3CA<br>8EXL	PIK3R1<br>7PG5
Known Inhibitors						
Sorafenib	--	--	--	-8.8	-8.7	-7
Alpelisib	--	--	--	-8.3	-8.4	-6.9
Tamoxifen	-6.0	--	--	--	--	--
Ganetespib	--	-8.5	--	--	--	--
Linsitinib	--	--	-9.2	--	--	--
Pre-bound ligands						
benzyl butyl benzene-1,2-dicarboxylate	-7.8	--	--	--	--	--
N,N-dimethyl-7H-purin-6-amine	--	-4.9	--	--	--	--
3-cyano-N-{1-[4-(5-cyano-1H-indol-3-yl)butyl]piperidin-4-yl}-1H-indole-7-carboxamide	--	--	-9.9	--	--	--
(4M)-4-{(4R)-3-[(2S)-2-methylbutyl][1,2,4]triazolo[4,3-a]pyridin-7-yl}-N-(1-methyl-1H-pyrazol-5-yl)pyrimidin-2-amine	--	--	--	-8.7	--	--
2-methyl-2-(4-{2-[3-methyl-1-(propan-2-yl)-1H-1,2,4-triazol-5-yl]-5,6-dihydroimidazo[1,2-d][1,4]benzoxazepin-9-yl}-1H-pyrazol-1-yl)propanamide	--	--	--	--	-10	--

Table S5. Binding affinities of OT bioactive compounds to hub proteins in BC.

Gene name<br>PDB ID	ESR1<br>4MG6	HSP90AB1<br>7ULJ	IGF1R<br>3LW0	MAPK1<br>8U8J	PIK3CA<br>8EXL	PIK3R1<br>7PG5
(-)-Catechin	-6.5	-7.3	-7.8	-8.6	-8.2	-5.9
(-)-Epicatechin	-8.0	-7.3	-7.7	-8.6	-7.4	-6.5
(-)-Epigallocatechin	-7.6	-7.4	-7.8	-8.7	-7.5	-6.4
(-)-Gallocatechin	-6.4	-7.7	-8.1	-8.4	-8.1	-5.8
(-)-Icariside B5	-7.1	-6.6	-7.4	-7.1	-7.3	-6.4
(+)-Catechin	-7.1	-7.2	-7.7	-8.7	-7.8	-6.1
(+)-Epicatechin	-7.6	-7.3	-8.2	-8.6	-8.1	-6.4
(+)-Gallocatechin	-7.2	-7.2	-7.7	-8.7	-7.8	-5.9
1-(3,4,5-trihydroxyphenyl)ethanone	-5.6	-5.3	-5.9	-6.3	-5.8	-5.3
Ascorbic acid	-5.3	-5.1	-5.2	-5.4	-4.8	-4.9
Caffeic acid	-6.0	-5.7	-6.3	-6.7	-6.6	-5.6
Catechol	-5.0	-4.7	-4.8	-5.3	-4.6	-4.6
Chakanoside I	-7.5	-7.3	-7.2	-7.3	-7.1	-6.0
Cyanidin	-8.2	-7.8	-7.8	-8.8	-8.2	-6.1
Delphinidin	-6.9	-7.6	-7.9	-8.8	-7.9	-5.9
Dihydroquercetin	-7.6	-7.8	-8.1	-8.5	-8.3	-6.3
Fisetin	-8.2	-7.5	-8.0	-8.6	-8.3	-5.9
Gallic acid	-5.4	-5.3	-5.8	-6.1	-5.6	-5.3
Leucocyanidin	-8.1	-7.3	-8.3	-8.8	-8.1	-5.9
Luteolin	-8.1	-7.9	-8.0	-9.1	-8.4	-6.3
Quercetin	-8.1	-7.7	-8.0	-8.8	-8.3	-6.2
Tricetinidin	-7.9	-7.5	-7.9	-8.8	-8.2	-6.0

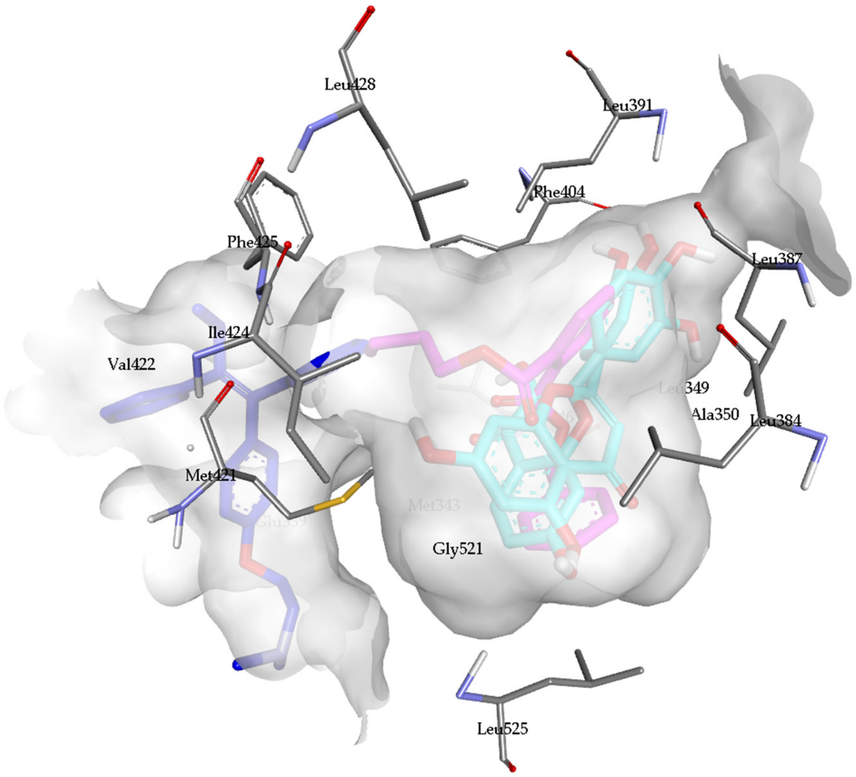

Figure S1. Superimposed 3D graphical representation of the control drug tamoxifen (shown in navy blue), prebound ligand benzyl butyl benzene-1,2-dicarboxylate (pink), and the top three ligands with the lowest binding affinities (cyanidin, fisetin, and luteolin; shown in cyan) bound to the protein ESR1, illustrating their spatial arrangement and potential binding conformations within the active site.

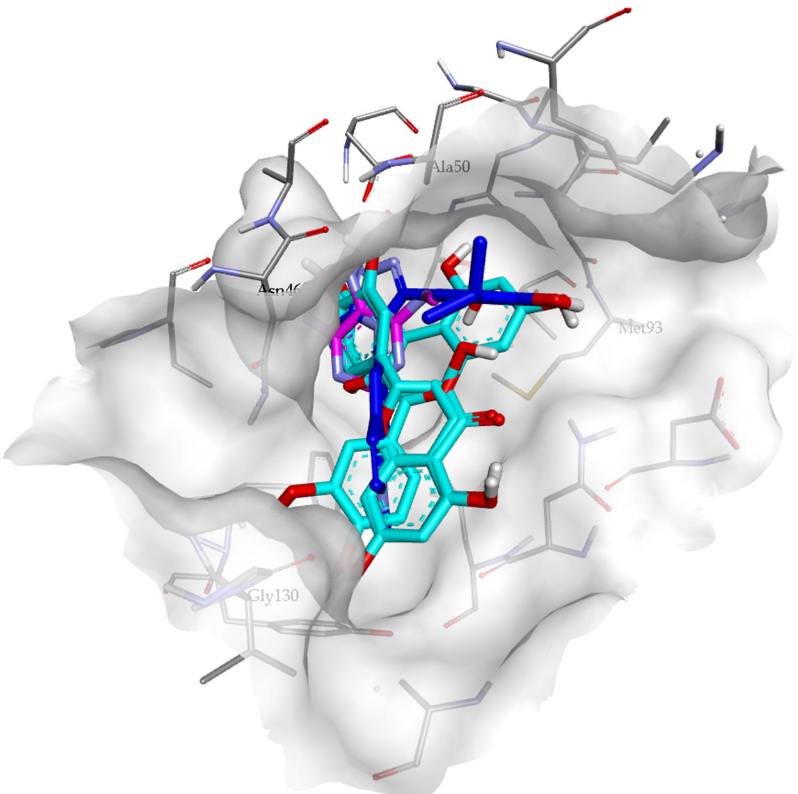

Figure S2. Superimposed 3D graphical representation of the control drug ganetespib (shown in navy blue), prebound ligand N,N-dimethyl-7H-purin-6-amine (pink), and the top three ligands with the lowest binding affinities (luteolin, dihydroquercetin, and quercetin; shown in cyan) bound to the protein HSP90AB1, illustrating their spatial arrangement and potential binding conformations within the active site.

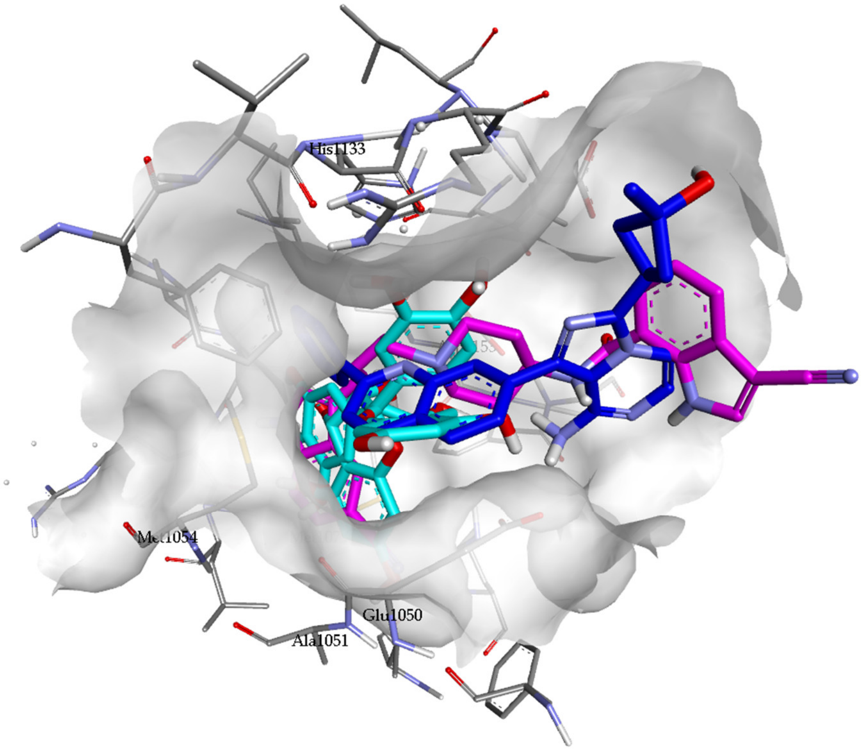

Figure S3. Superimposed 3D graphical representation of the control drug linsitinib (shown in navy blue), prebound ligand 3-cyano-N-{1-[4-(5-cyano-1H-indol-3-yl)butyl]piperidin-4-yl}-1H-indole-7-carboxamide (pink), and the top three ligands with the lowest binding affinities (leucocyanidin, (+)-epicatechin, and (-)-gallocatechin; shown in cyan) bound to the protein IGF1R, illustrating their spatial arrangement and potential binding conformations within the active site.

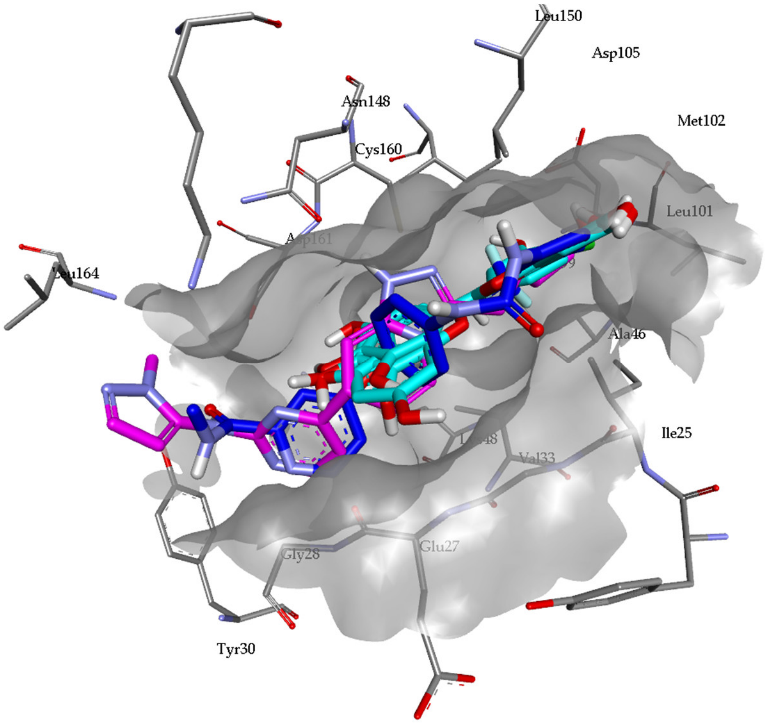

Figure S4. Superimposed 3D graphical representation of the control drug sorafenib (shown in navy blue), prebound ligand (4M)-4-{(4R)-3-[(2S)-2-methylbutyl][1,2,4]triazolo[4,3-a]pyridin-7-yl}-N-(1-methyl-1H-pyrazol-5-yl)pyrimidin-2-amine (pink), and the top three ligands with the lowest binding affinities (fisetin, luteolin, and quercetin; shown in cyan) bound to the protein MAPK1, illustrating their spatial arrangement and potential binding conformations within the active site.

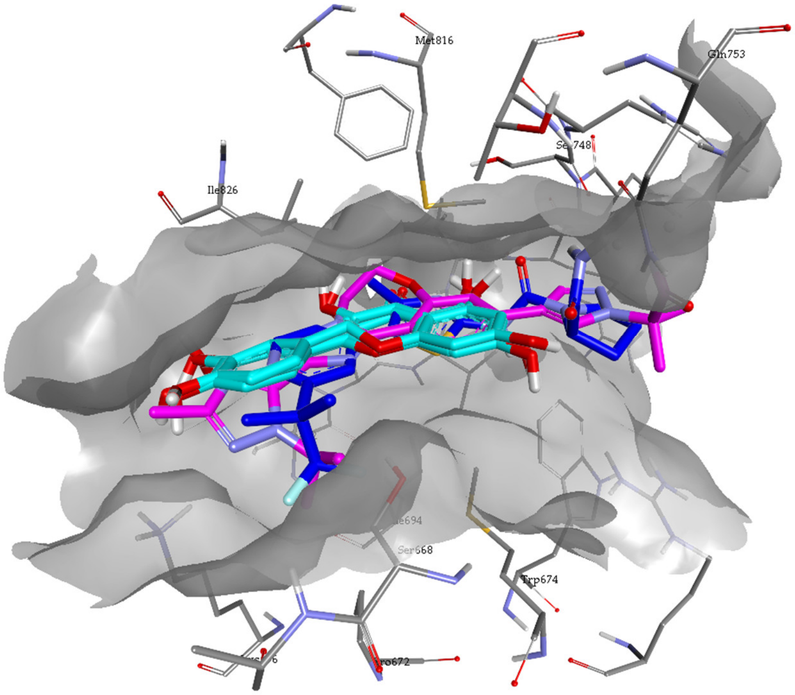

Figure S5. Superimposed 3D graphical representation of the control drug alpelisib (shown in navy blue), prebound ligand 2-methyl-2-(4-{2-[3-methyl-1-(propan-2-yl)-1H-1,2,4-triazol-5-yl]-5,6-dihydroimidazo[1,2-d][1,4]benzoxazepin-9-yl}-1H-pyrazol-1-yl)propenamide (pink), and the top three ligands with the lowest binding affinities (luteolin, quercetin, and dihydroquercetin; shown in cyan) bound to the protein PIK3CA, illustrating their spatial arrangement and potential binding conformations within the active site.

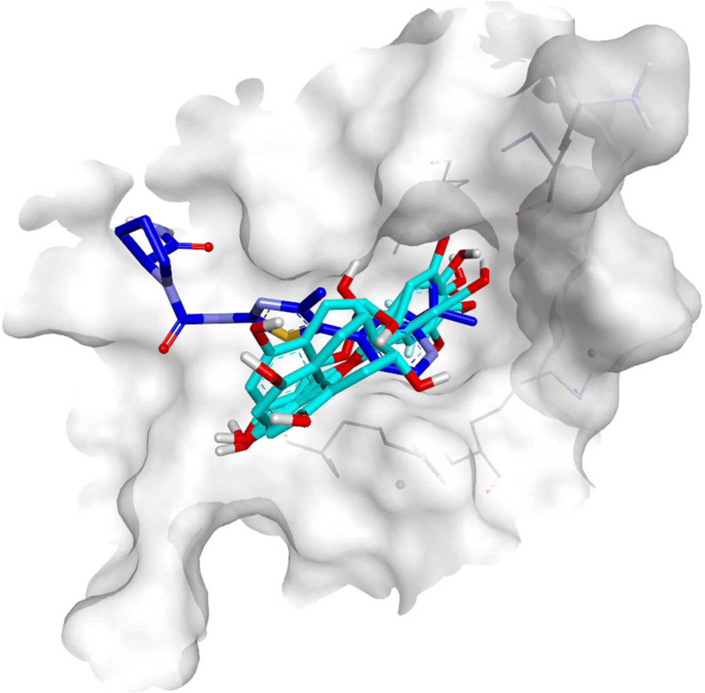

Figure S6. Superimposed 3D graphical representation of the control drug alpelisib (shown in navy blue) and the top three ligands with the lowest binding affinities ((-)-epicatechin, (-)-epigallocatechin, and (+)-epicatechin; shown in cyan) bound to the protein PIK3R1, illustrating their spatial arrangement and potential binding conformations within the active site.

Table S6. Comparative analysis of residue flexibility changes in MAPK1 and PIK3CA between apo and holo states upon luteolin binding.

Residue no.	Residue Name	Apo state	Holo state	% change
		MAPK1		
179	Threonine	3.206	1.514	-52.776
35	Glycine	1.92	0.908	-52.7083
178	Histidine	2.986	1.423	-52.3443
34	Tyrosine	2.257	1.081	-52.1046
185	Tyrosine	2.466	1.183	-52.0276
36	Methionine	1.577	0.833	-47.1782
184	Glutamic Acid	2.319	1.243	-46.3993
37	Valine	1.286	0.712	-44.6345
30	Glycine	1.941	1.077	-44.5131
31	Glutamic Acid	2.076	1.172	-43.5453
29	Isoleucine	1.687	0.967	-42.6793
202	Glycine	2.066	1.185	-42.6428
180	Glycine	2.85	1.637	-42.5614
33	Alanine	2.421	1.411	-41.7183
177	Aspartic Acid	2.921	1.729	-40.8079
32	Glycine	2.144	1.304	-39.1791
38	Cysteine	1.073	0.679	-36.7195
12	Valine	2.024	1.287	-36.413
176	Histidine	3.283	2.091	-36.3083
17	Phenylalanine	1.646	1.06	-35.6015
28	Tyrosine	1.538	0.996	-35.2406
18	Aspartic Acid	1.574	1.026	-34.8158
19	Valine	1.44	0.944	-34.4444
13	Arginine	2.398	1.578	-34.1952
16	Valine	2.081	1.379	-33.7338
199	Asparagine	1.764	1.18	-33.1066
11	Methionine	2.154	1.447	-32.8227
197	Methionine	1.437	0.971	-32.4287
14	Glycine	2.634	1.789	-32.0805
117	Glutamine	1.285	0.879	-31.5953
15	Glutamine	2.356	1.622	-31.1545
201	Lysine	1.764	1.217	-31.0091
336	Leucine	1.69	2.638	56.09467
341	Leucine	1.299	1.979	52.34796
337	Proline	1.744	2.597	48.91055
338	Lysine	1.646	2.433	47.81288
340	Lysine	1.474	2.173	47.42198
339	Glutamic Acid	1.587	2.277	43.47826
322	Isoleucine	2.085	2.974	42.63789
249	Aspartic Acid	1.166	1.662	42.53859
248	Glutamic Acid	1.269	1.806	42.31678
344	Leucine	1.101	1.536	39.50954
247	Glutamine	1.235	1.716	38.94737
250	Leucine	1.192	1.649	38.33893
146	Arginine	0.793	1.094	37.95712

342	Lysine	1.248	1.712	37.17949
294	Phenylalanine	0.608	0.829	36.34868
246	Serine	1.118	1.524	36.31485
335	Aspartic Acid	2.056	2.789	35.65175
264	Serine	1.758	2.334	32.76451
296	Proline	0.678	0.899	32.59587
295	Asparagine	0.652	0.864	32.51534
208	Aspartic Acid	0.497	0.658	32.39437
145	Histidine	0.712	0.942	32.30337
324	Glutamic Acid	1.479	1.951	31.91346
251	Asparagine	1.387	1.817	31.00216
343	Glutamic Acid	1.234	1.614	30.79417
345	Isoleucine	0.961	1.25	30.07284
**PIK3CA**				
469	Asparagine	1.8	0.728	-59.5556
410	Arginine	3.593	1.496	-58.3635
468	Tryptophan	1.491	0.656	-56.0027
409	Asparagine	3.717	1.737	-53.2688
408	Serine	4.142	2.091	-49.5171
945	Tryptophan	2.611	1.322	-49.3681
470	Serine	1.558	0.823	-47.1759
829	Glycine	1.66	0.893	-46.2048
828	Phenylalanine	1.42	0.766	-46.0563
407	Leucine	5.056	2.838	-43.8687
944	Glycine	2.605	1.468	-43.6468
833	Aspartic Acid	1.915	1.089	-43.1332
467	Lysine	1.172	0.676	-42.3208
126	Methionine	5.071	2.927	-42.2796
125	Serine	3.933	2.271	-42.2578
411	Leucine	2.88	1.673	-41.9097
139	Glutamic Acid	5.332	3.153	-40.8665
832	Leucine	1.557	0.927	-40.4624
501	Proline	0.981	0.587	-40.1631
502	Aspartic Acid	0.952	0.578	-39.2857
570	Histidine	1.762	1.07	-39.2736
326	Tyrosine	1.161	0.71	-38.8458
256	Histidine	1.053	0.66	-37.3219
536	Glutamic Acid	0.848	0.534	-37.0283
327	Threonine	1.206	0.766	-36.4842
257	Glycine	1.319	0.839	-36.3912
312	Glutamic Acid	2.671	1.699	-36.3909
498	Cysteine	1.136	0.724	-36.2676
499	Asparagine	1.076	0.687	-36.1524
313	Histidine	2.306	1.49	-35.3859
140	Tyrosine	4.456	2.881	-35.3456
311	Glutamic Acid	3.093	2.002	-35.2732
535	Tyrosine	0.916	0.593	-35.262
314	Cysteine	2.085	1.35	-35.2518
406	Glycine	6.209	4.046	-34.8365

353	Isoleucine	1.496	0.977	-34.6925
831	Phenylalanine	1.445	0.946	-34.5329
506	Arginine	0.89	0.587	-34.0449
403	Serine	6.277	4.145	-33.9653
351	Asparagine	1.398	0.924	-33.9056
350	Leucine	1.53	1.013	-33.7908
827	Aspartic Acid	1.207	0.8	-33.72
352	Proline	1.392	0.924	-33.6207
202	Serine	3.709	2.465	-33.54
349	Leucine	1.741	1.161	-33.3142
310	Lysine	3.66	2.441	-33.306
255	Tyrosine	1.014	0.677	-33.2347
830	Histidine	1.64	1.095	-33.2317
471	Arginine	1.202	0.803	-33.1947
315	Proline	1.853	1.24	-33.0815
302	Serine	1.989	1.332	-33.0317
249	Tyrosine	1.753	1.174	-33.0291
943	Glycine	2.062	1.388	-32.6867
301	Cysteine	1.76	1.185	-32.6705
835	Lysine	2.387	1.608	-32.6351
243	Arginine	3.644	2.456	-32.6015
497	Aspartic Acid	1.016	0.686	-32.4803
496	Leucine	0.895	0.605	-32.4022
537	Glutamine	0.874	0.591	-32.3799
254	Isoleucine	1.049	0.71	-32.3165
266	Asparagine	1.676	1.138	-32.1002
503	Proline	0.954	0.648	-32.0755
253	Glycine	1.146	0.781	-31.8499
241	Asparagine	3.771	2.574	-31.7422
267	Threonine	1.718	1.18	-31.3155
248	Isoleucine	1.95	1.341	-31.2308
811	Histidine	1.345	0.928	-31.0037
264	Asparagine	1.507	1.041	-30.9224
250	Valine	1.51	1.045	-30.7947
354	Glycine	1.334	0.927	-30.5097
834	Histidine	2.207	1.534	-30.4939
845	Arginine	1.345	0.939	-30.1859
507	Glycine	0.922	0.644	-30.1518
348	Aspartic Acid	1.98	1.383	-30.1515
127	Leucine	6.309	4.407	-30.1474
75	Isoleucine	0.927	2.327	151.0248
74	Histidine	1.043	2.586	147.9386
72	Proline	1.011	2.439	141.2463
64	Asparagine	0.667	1.564	134.4828
73	Lysine	1.102	2.567	132.9401
76	Tyrosine	0.971	2.222	128.8363
71	Leucine	1.028	2.303	124.0272
70	Glutamic Acid	1.081	2.299	112.6735
172	Methionine	0.924	1.951	111.1472
77	Asparagine	1.159	2.439	110.44

171	Isoleucine	0.825	1.718	108.2424
173	Leucine	1.003	2.071	106.4806
903	Glycine	1.292	2.658	105.7276
170	Cysteine	0.743	1.512	103.4993
65	Valine	0.691	1.406	103.4732
78	Lysine	1.221	2.452	100.819
174	Glycine	1.051	2.08	97.90676
67	Serine	0.998	1.943	94.68938
69	Proline	1.255	2.422	92.98805
79	Leucine	1.145	2.207	92.75109
175	Arginine	0.961	1.842	91.67534
169	Serine	0.761	1.444	89.75033
68	Serine	1.132	2.146	89.57597
66	Glutamic Acid	0.799	1.491	86.60826
80	Aspartic Acid	1.373	2.548	85.57902
168	Arginine	0.747	1.382	85.00669
176	Methionine	0.875	1.611	84.11429
81	Lysine	1.515	2.781	83.56436
904	Methionine	1.341	2.454	82.99776
82	Glycine	1.288	2.325	80.51242
83	Glutamine	1.069	1.892	76.98784
162	Serine	0.802	1.413	76.18454
177	Proline	0.715	1.258	75.94406
167	Isoleucine	0.676	1.173	73.52071
108	Aspartic Acid	1.156	1.991	72.23183
192	Proline	1.155	1.952	69.00433
107	Histidine	1.229	2.045	66.39544
161	Leucine	0.77	1.278	65.97403
178	Asparagine	0.669	1.075	60.68759
164	Tyrosine	0.674	1.082	60.53412
193	Methionine	1.101	1.753	59.21889
767	Asparagine	1.781	2.832	59.01179
163	Glutamine	0.791	1.257	58.91277
902	Serine	1.313	2.073	57.88271
109	Cysteine	0.984	1.55	57.52033
768	Serine	1.442	2.252	56.17198
166	Tyrosine	0.689	1.075	56.02322
84	Isoleucine	1.023	1.596	56.01173
86	Valine	0.828	1.291	55.91787
106	Asparagine	1.086	1.691	55.70902
165	Lysine	0.706	1.088	54.10765
906	Glutamic Acid	1.215	1.866	53.58025
87	Valine	0.76	1.164	53.15789
85	Isoleucine	0.981	1.497	52.59939
104	Lysine	1.06	1.608	51.69811
722	Glycine	0.866	1.304	50.57737
160	Proline	0.884	1.32	49.32127
63	Proline	0.73	1.09	49.31507
62	Proline	0.651	0.965	48.23349
61	Tyrosine	0.621	0.909	46.37681

103	Leucine	0.985	1.429	45.07614
905	Proline	1.444	2.092	44.87535
105	Isoleucine	1.097	1.566	42.75296
1	Asparagine	3.535	5.043	42.65912
102	Threonine	1.009	1.437	42.41824
179	Leucine	0.666	0.946	42.04204
110	Valine	0.94	1.319	40.31915
721	Glutamine	0.888	1.245	40.2027
793	Arginine	0.704	0.986	40.05682
719	Glutamine	0.796	1.107	39.07035
864	Glutamic Acid	1.79	2.489	39.05028
723	Leucine	0.807	1.122	39.03346
725	Leucine	0.744	1.032	38.70968
686	Leucine	0.858	1.188	38.46154
159	Tyrosine	0.927	1.28	38.07983
184	Lysine	1.003	1.384	37.98604
194	Aspartic Acid	1.048	1.446	37.9771
149	Cysteine	0.683	0.942	37.92094
794	Serine	0.657	0.903	37.44292
720	Asparagine	0.891	1.218	36.70034
191	Leucine	1.197	1.627	35.92314
718	Tryptophan	0.705	0.958	35.88652
687	Leucine	0.795	1.077	35.4717
148	Valine	0.647	0.875	35.23957
865	Cysteine	1.719	2.322	35.07853
790	Leucine	0.833	1.114	33.73349
111	Proline	0.869	1.162	33.71692
186	Serine	1.257	1.68	33.65155
183	Alanine	0.956	1.276	33.4728
724	Aspartic Acid	0.863	1.139	31.98146
195	Cysteine	1.045	1.379	31.96172
113	Glutamine	1.039	1.363	31.18383
769	Histidine	1.378	1.806	31.05951
60	Valine	0.631	0.825	30.74485
791	Phenylalanine	0.751	0.981	30.62583
101	Tyrosine	0.996	1.299	30.42169
792	Threonine	0.738	0.962	30.3523
789	Aspartic Acid	0.918	1.195	30.17429
907	Leucine	1.134	1.476	30.15873

Table S7. Residue flexibility changes in binding site-interactive amino acids of MAPK1 and PIK3CA upon luteolin binding.

Residue no.	Residue Name	Apo state	Holo state	% change
		MAPK1		
103	Glutamine	0.767	0.663	-13.5593
103	Glutamine	1.286	0.712	-44.6345
37	Valine	0.844	0.632	-25.1185
52	Lysine	0.721	0.667	-7.4896

164	Cysteine	0.684	0.588	-14.0351
154	Leucine	1.687	0.967	-42.6793
29	Isoleucine	0.786	0.66	-16.0305
106	Methionine	0.748	0.726	-2.94118
165	Aspartic Acid	0.833	0.716	-14.0456
109	Aspartic Acid	0.767	0.663	-13.5593
		PIK3CA		
730	Tyrosine	0.644	0.7	8.695652
694	Isoleucine	0.638	0.73	14.42006
745	Valine	0.993	0.963	-3.02115
744	Valine	0.757	0.753	-0.5284
816	Methionine	0.969	0.888	-8.45361
826	Isoleucine	0.944	0.773	-18.1144
742	Isoleucine	0.591	0.669	13.19797
704	Aspartic Acid	0.744	0.66	-11.2903

Table S8. Thermodynamic profiles of the luteolin binding onto the hub BC proteins based on MMPBSA. All values are expressed in kcal/mol.

**Complex**	**Time Frame (ns)**	**ΔH (kcal/mol)**							**-TΔS**	**ΔG**
		VDW	***EEL***	***EPB***	***ENPOLAR***	***GGAS***	***GSOLV***	***TOTAL***		
PIK3CA	50 – 70 ns	-21.26	-26.84	33.04	-2.73	-48.1	30.31	-17.79	9.44	-8.35
MAPK1	100 – 120 ns	-32.54	-34.14	47.19	-3.46	-66.68	43.73	-22.94	7.42	-15.52

Notes: VDW, van der Waals energy; EEL, electrostatic energy; EPB, MMPBSA polar solvation energy; ENPOLAR, MMPBSA non-polar solvation energy; GGAS, net gas phase energy; GSOLV, net solvation energy; TOTAL, total *ΔH*
